# Microtubule-associated protein 4 forms aggregates consisting of helical filaments

**DOI:** 10.1016/j.bbrep.2025.102118

**Published:** 2025-06-27

**Authors:** Shuto Miura, Eisuke Ishibashi, Takuto Nakamichi, Koji Uwai, Masahiro Kuragano, Kiyotaka Tokuraku

**Affiliations:** Graduate School of Engineering, Muroran Institute of Technology, Hokkaido, 050-8585, Japan

**Keywords:** Amyloid fibril, Microtubule-associated protein 4, Myocardial infarction, Protein aggregation, Quantum dot, Tau

## Abstract

We previously reported that microtubule-associated protein (MAP) 4 was detected in the cytoplasm as abnormal “puncta” in post-ischemic mouse cardiomyocytes. MAP4, a member of the MAP superfamily, has a repeat region consisting of multiple microtubule-binding sequences in its microtubule-binding domain (MBD), like tau. The tau forms aggregates composed of amyloid fibrils and grows into neurofibrillary tangles in neurons. Therefore, we hypothesized that MAP4 also forms amyloid fibrils in cells. Here, we observed whether MAP4 forms aggregates composed of amyloid fibrils using fluorescence microscopy and transmission electron microscopy with quantum dot (QD) nanoprobes. Since we had previously succeeded in real-time 3D imaging of tau MBD fragment aggregate formation using QD nanoprobes, we attempted to observe aggregates using human MAP4 MBD fragments under the same conditions. Fluorescence microscopy showed that 10 μM MAP4 formed aggregates at a rate similar to that of tau. Time-laps 3D imaging by confocal laser microscopy revealed that MAP4 aggregate grains were smaller in size and the deposits were thinner than tau aggregates. Transmission electron microscopy of the MAP4 aggregates revealed that they consisted of helical filaments with a width of 22.6 ± 2.8 nm and a helical pitch length of 48.2 ± 8.4 nm. The helical filaments of MAP4 were shorter in width and longer in helical pitch than those of tau. Furthermore, MAP4 aggregates did not increase the fluorescence intensity of thioflavin T (ThT), and the circular dichroism (CD) spectrum slightly differed from that of tau. These findings suggest that while MAP4 forms aggregates composed of helical filaments similar to those of tau, the structural properties of these filaments are somewhat distinct.

## Highlights

• MAP4 formed aggregates consisting of helical filaments.
• Aggregation rates of MAP4 and tau were comparable.
• MAP4 aggregates were smaller and their deposits were thinner than those of tau.
• The structural properties of MAP4 filaments differed somewhat from those of tau.

## Graphical abstract

**Figure:**
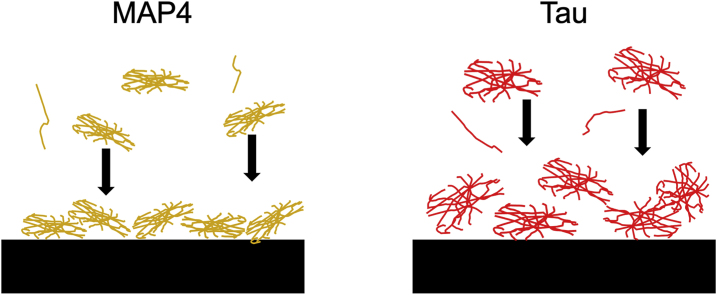

## 1 Introduction

The myocardium is a muscular tissue of the heart that possesses distinctive physiological, mechanical, microstructural, and electrical characteristics, and is a paradigm of functional efficiency [1]. Myocardial infarction is a leading cause of death and disability worldwide [2]. A decline in cardiac function after myocardial infarction has been reported as a leading cause of chronic heart failure, with systemic health complications and increased mortality [3]. There are no effective treatments to improve the contractility of cardiomyocytes because currently available inotropic therapies are associated with high morbidity and mortality in patients with systolic heart failure [4,5] and have shown a very modest risk reduction [6]. A microtubule-associated protein (MAP), MAP4, was shown to increase significantly in the hearts of individuals with cardiomyopathy, which is a cause of chronic heart failure [7]. Recently, we demonstrated an increase in the amount of both MAP4 and phosphorylated MAP4 by microtubule affinity-regulated kinase 4 (MARK4) in mouse cardiomyocytes after ischemia [8]. Furthermore, MAP4 was detected as abnormal “puncta” in the cardiomyocytes of post-ischemic mice [8]. However, details of the structure and formation of MAP4 puncta are still unknown.

Microtubules are part of the cytoskeleton and are involved in the maintenance of cell morphology, cellular deformation, and intracellular transport [9,10]. MAPs are a family of structurally similar proteins that bind to the surface of microtubules and promote and stabilize microtubule polymerization [9,10]. MAP4, a microtubule-binding protein, is widely present in all cells [9,10]. MAP4 has a microtubule-binding domain (MBD) at its C-terminus, which contains three to five tubulin-binding repeat sequences [11]. Different numbers of repeat sequences formed by alternative splicing differentially affected protein activity, and the distribution of these proteins affected basic physiological phenomena in cells, primarily microtubule bundling [11]. MAP2 and tau, which belong to the same superfamily as MAP4, are localized in neurons, and isoforms with different numbers of tubulin-binding repeats exist, like MAP4 [10]. A molecular phylogenetic analysis of isoforms of these MAPs revealed that the primary structures of the repeat regions of MAP4 and tau were more closely related than those of MAP2 [12].

**Fig. 1:**
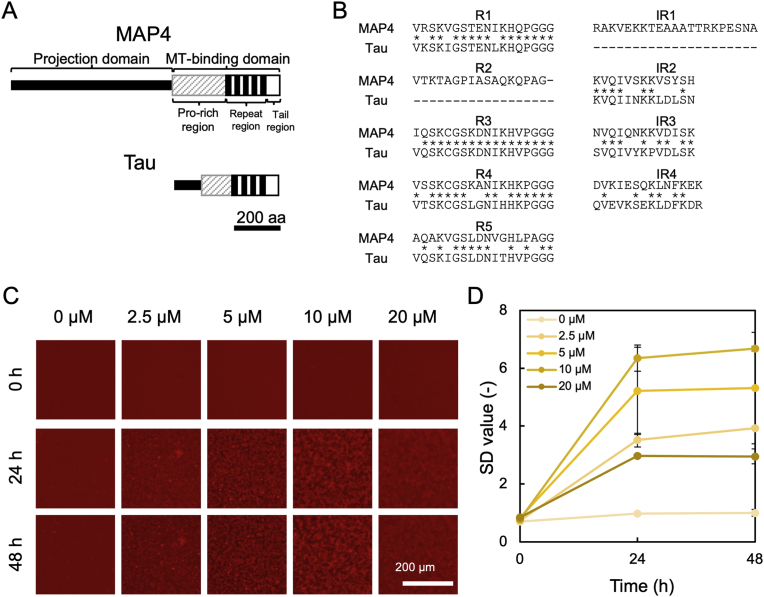
MAP4 aggregation in the buffer condition for tau aggregation containing heparin and DTT. (A) Schematic structures of MAP4 and tau superfamily proteins [12]. The structures are classified into a projection domain and a microtubule-binding domain (MBD). MBDs are further separated into Pro-rich, repeat, and tail regions. The repeat region consists of tandemly repeated AP sequences shown as black boxes. The part used in this experiment is the MT-binding domain. (B) Comparison of amino acid sequences of repeat regions of MAP4 and tau, which were used in this study. R1 to R5 show the repeat sequences of MAP4. IR1 to IR5 show the Inter-repeat sequence. ∗ indicates conserved amino acids in the primary structures of MA4 and tau. (C) Sequential images of the process of MAP4 aggregation at the indicated concentration. Images were captured every 24 h using conventional fluorescence microscopy for 48 h. (D) SD value at each time point was quantified using Image J software. Data represent mean values, and error bars indicate SD derived from three separate experiments.

Previously, we successfully observed the process of amyloid β aggregate formation in real-time using quantum dot (QD)-labeled amyloid β [13,14]. QDs, which are fluorescent semiconductor nanoparticles, exhibit unique optical properties that are inherent to their nanocrystalline structure [13]. QDs are useful for real-time analysis of protein dynamics over long periods due to their chemical and physical stability, and they display long-term photostability [13]. Using these advantages, we observed the aggregation process of tau MBD fragment, an amyloid protein that causes Alzheimer's disease [[15], [16], [17]]. In addition, QD imaging technology has been successfully used for real-time imaging of various amyloid proteins and can be applied to the visualization of a wide range of aggregating proteins [[17], [18], [19]]. Tau, which we previously successfully visualized, has a repeat region that is highly homologous to MAP4 [12] (Fig. 1A and B). Tau forms amyloid aggregates in neurons of Alzheimer's disease (AD) patients [16,17]. It has also been reported that the sequence of VQIVYK in IR3 of Tau in Fig. 1B is an aggregation-prone sequence [20], as VQIVSK in IR2 of MAP4 in Fig. 1B has a similar sequence to the aggregation-affected sequence, it is quite possible that MAP4, which has a repeat region, like tau, also forms amyloid aggregates.

In this study, we attempted to clarify whether the MAP4 MBD fragment forms aggregates *in vitro* using fluorescence microscopy and confocal laser microscopy with QD imaging techniques. The results revealed that MAP4 fragment aggregates formed at a similar rate to tau, but the grain size of the aggregates was smaller than that of tau. Furthermore, these aggregates were composed of amyloid-like fibrils with a helical structure like tau, although the fibril width, helix pitch length, and sensitivity to ThT were somewhat different from those of tau. We believe that our findings provide new insight into the field of protein aggregation and contribute to the elucidation of the pathology of heart failure.

## 2 Materials and methods

### 2.1 Construction of expression plasmids for human MAP4 and tau MBD fragments

The full-length cDNA of human MAP4 was purchased from Danaform Corporation (Clone ID: H013078A07). To construct expression plasmid for the MAP4 MBD, a primer set for the MAP4 gene (MAP4-forward; 5′-AGACATATGCCACCAAGCCCAGAGAAG-3′, MAP4-reverse; 5′-CCCCTCGAGTTAGATGCTTGTCTCCTGGAT-3′). These primers contain *Nde*I and *Xho*I restriction sites and were purchased from Hokkaido System Science Co., Ltd. Human MBD MAP4 was amplified by PCR using PrimeSTAR®Max DNA Polymerase (TaKaRa Bio). PCR was performed under the following conditions: 98 °C for 10 s for heat denaturation, 58 °C for 5 s for annealing, and 72 °C for 8 s for primer extension, repeated for 30 cycles. The purified PCR products were digested at both ends using restriction enzymes *Nde*I (TaKaRa Bio) and *Xho*I (TaKaRa Bio) and introduced into pET-21a(+) vector.

The full-length cDNA of human tau was purchased from Addgene (Clone ID: 87368). To construct expression plasmid for the human tau MBD, a primer set for the human tau gene (human tau-forward; 5′-AGACATATGGCTGAGCCCCGCCAGGA-3′, human tau-reverse; 5′-CCCTCGAGTCACAAACCCTGCTTGGC-3′). These primers contain *Nde*I and *Xho*I restriction sites and were purchased from Hokkaido System Science Co., Ltd. Human MBD tau was amplified by PCR using PrimeSTAR®Max DNA Polymerase. PCR was performed under the following conditions: 98 °C for 10 s for denaturation, 55 °C for 5 s for annealing, and 72 °C for 8 s for primer extension, repeated for 30 cycles. The purified PCR products and DNA were digested at both ends using restriction enzymes *Nde*I and *Xho*I and introduced into pET-21a(+) vector.

The nucleotide sequence of constructed plasmids was confirmed by DNA sequencing, which was outsourced to Hokkaido System Science Co., Ltd.

### 2.2 Purification of human MAP4 and tau MBD fragments

Bacterial expression and purification of human MBD MAP4 and human MBD tau were performed according to previously reported methods [11,21,22]. Briefly, the expression plasmid was transformed into *Escherichia coli* (Rosetta (DE3) pLys), and protein expression was induced with 1 mM isopropyl-1-thio-β-d-galactopyranoside. The thermostable fractions of each extract were subjected to sequential column chromatography using a UNOsphere™ S column (Bio-Rad Laboratories Inc.) and a TOYOPEARL® Butyl-650 column (Tosoh Co., Ltd.). In the UNOsphere™ S and TOYOPEARL® Butyl-650 column chromatography, the bound proteins were eluted using a gradient of 0–1 M NaCl and 1.2 M − 0 M (NH_4_)_2_SO_4_, followed by dialysis. SDS-PAGE [23] confirmed the purity of the purified proteins, and the concentration was evaluated using the Lowry method [24].

### 2.3 Observation of MAP4 aggregates using QD

MAP4 solutions were prepared by mixing 0, 2.5, 5, 10, and 20 μM MAP4, 50 nM QD, 10 μM heparin, 10 mM DTT, and 1 × Phosphate-buffered saline (PBS); MAP4 solutions were dispensed into 1536 well plates, centrifuged (5 min, 3700 rpm), and incubated at 37 °C. Each well was then observed over time with an inverted fluorescence microscope (Nikon, TE2000 with 4 × lens) equipped with a CCD camera (DP72, Olympus) and a confocal laser microscope (Nikon, Nikon C2 Plus).

### 2.4 Transmission electron microscope observation

MAP4 solution was prepared by mixing 10 μM MAP4 or 10 μM tau, 0 and 50 nM QD, 0 and 10 μM heparin, 10 mM DTT, 1 × PBS, 37 °C, and after incubating for 24 h, 10 μL was placed on a collodion support membrane (No. U1005, EM Japan K.K.) and incubated at room temperature for 5 min. The grids were washed twice with ultrapure water and treated with two doses of 0.5 % phosphotungstic acid. The grids were dried thoroughly at room temperature for 5 min before observation with an LVEM5 low-voltage transmission electron microscope (Delong) and an H-7600 transmission electron microscope (Hitachi).

### 2.5 Thioflavin T fluorescence experiment

Samples were prepared by mixing 10 μM MAP4 or 10 μM tau, 20 μM thioflavin T (ThT), 10 μM heparin, 10 mM DTT, and 1 × PBS. Samples were aliquoted into 384-well plates, centrifuged (5 min, 3700 rpm), and incubated at 37 °C. Over time, each well was measured with a Corona microplate reader (SH-9000Lab).

### 2.6 Circular dichroism spectroscopy

The changes in the secondary structures of Aβ_42_, tau, and MAP4 during the 24 h aggregation process were monitored using circular dichroism (CD) spectroscopy. Samples were prepared by mixing 10 μM tau or 10 μM MAP4, 10 μM heparin, 10 mM DTT, and 1 × PBS and were incubated at 37 °C for 24 h. As a control sample, 20 μM human Aβ_42_ (#4349-v; Peptide Institute) in PBS was incubated at 37 °C for 24 h. Measurements were performed using a J-720WI Spectropolarimeter (JASCO) equipped with a quartz micro sampling disk cell (MSD-462, path length: 0.2 mm). Scanning was conducted over the range of 185–450 nm with a bandwidth of 1 nm, and each sample was measured 10 times. To quantify the secondary structure, the proportion of each structure was calculated using the BeStSel web server (version 1.3.230210) [[25], [26], [27]], which predicts secondary structure content from CD spectra.

## 3 Results and discussion

### 3.1 The formation of MAP4 and tau aggregates

**Fig. 2:**
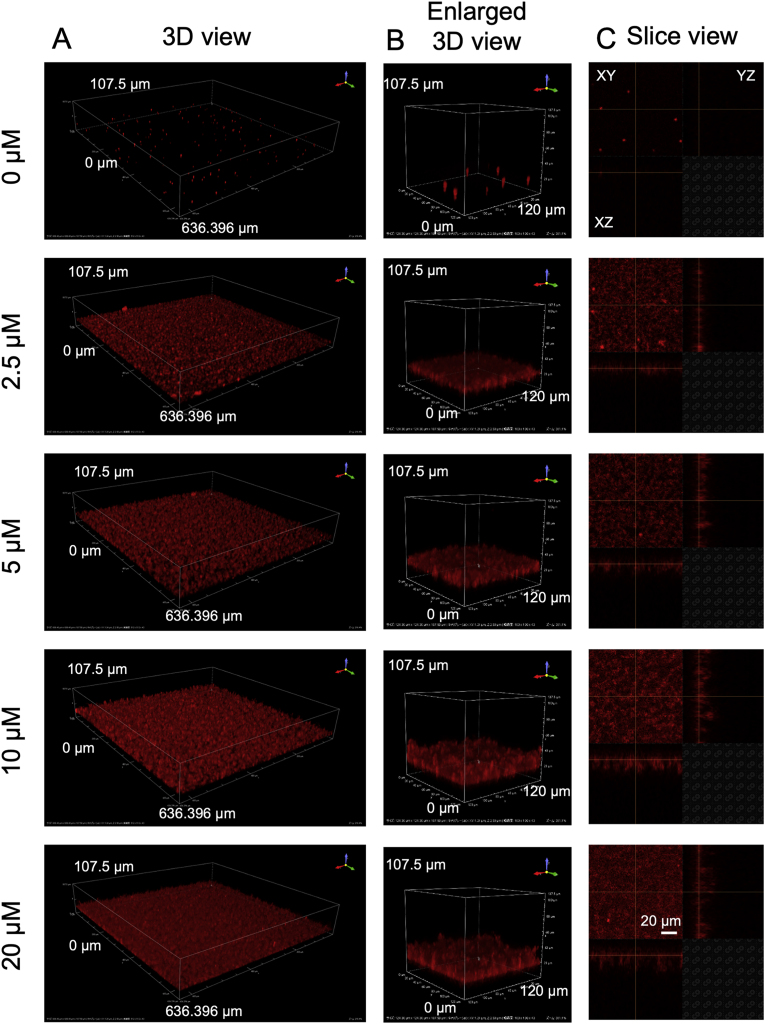
MAP4 aggregation under the buffer condition for tau aggregation. (A) 3D reconstruction images of MAP4 aggregates at the indicated concentration after 24 h incubation. (B) Enlarged 3D images of aggregates of each sample in panel (A). (C) Slice images of aggregates of each sample in panel (B).

First, MBD fragments of human MAP4 and tau were expressed in *Escherichia coli* (Rosetta 2 (DE3) pLys) and purified (Supplementary methods, Fig. S1, and Fig. S2) according to our previous study [12]. Using these protein fragments, we first attempted QD imaging to clarify whether MAP4 forms aggregates. MAP4 concentration and buffer conditions were set based on the experimental conditions in which the formation of tau aggregates was previously observed [16]. Specifically, the buffer was supplemented 10 μM heparin as an aggregation promoter and 10 mM DTT as a reducer of disulfide bonds in the protein molecules. 2.5, 5, 10, and 20 μM MAP4 were incubated at 37 °C and observed every 24 h for a total of 48 h with an inverted fluorescence microscope. As a result, the formation of aggregates was confirmed at 24 when MAP4 concentration was 2.5, 5, 10, and 20 μM, and no changes were observed between 24 h and 48 h (Fig. 1A). We then measured the standard deviation (SD) of brightness values per pixel from the fluorescent images, has these have been reported to be correlated with the amount of protein aggregates [28]. As shown in Fig. 1B, at 24 h, the SD value increased in a concentration-dependent manner when MAP4 concentrations were below 10 μM, indicating that the amount of aggregates had increased. The changes in the amount of aggregates became saturated at 24 h in all concentrations of MAP4. The three-dimensional observation using confocal laser microscopy (Fig. 2) suggested that the thickness of the deposited aggregates increased MAP4 concentration of increased, up to 10 μM because thickness did not increase with 20 μM MAP4. Interestingly, the view of the 3D volume demonstrated that the morphology and density of MAP4 aggregates were considerably different between 10 μM and 20 μM (Fig. 2). The fineness of aggregate structure also differed between both concentrations, suggesting that the presence of an abundance of MAP4 molecules may increase the nucleation of aggregates that grow to a size sufficient for deposition. Recently, we reported that in glycerol solutions of physiological clays, Aβ aggregates exhibit very fine and high density [29]. It makes sense that the same protein can exhibit different morphologies depending on the conditions under which it is aggregated, and it is possible that a similar reaction to that described above is occurring at higher protein concentrations. Here, we confirmed that MAP could form aggregates by mixing 2.5–20 μM MAP4, 50 nM QD, 10 μM heparin, 10 mM DTT, and 1 × PBS and incubating the mixture at 37 °C for 24 h. Furthermore, since the SD value increased in a concentration-dependent manner below 10 μM and became saturated at 24 h, subsequent quantitative evaluation of MAP4 aggregation was performed at a concentration of 10 μM for 24 h.

### 3.2 Effect of heparin and DTT on MAP4 aggregation

To understand the effect of heparin and DTT on MAP4 aggregation, we examined whether MAP4 aggregates formed, even in the absence of heparin and DTT. As a result, MAP4 aggregates could be observed only in the presence of heparin and DTT (Supplementary Fig. S3A) like tau [16]. As shown in Supplementary Fig. S3B, SD value increased only in the condition with heparin and DTT, and that had been saturated for 24 h. The observation with confocal laser microscopy at 24 h showed that aggregates formed in the presence of both heparin and DTT (Supplementary Fig. S3C).

It is well known that DTT is able to reduce disulfide bonds in proteins [30]. These disulfide bonds between cysteines in and between polypeptides maintain the tertiary structure of proteins [31]. The human MAP4 MBD fragment has two cysteines in the repeat region and one in the tail region. MAP4 may form disulfide bonds among these cysteine residues, which could hinder the formation of the β-sheet structures necessary for amyloid fibril formation. It has also been hypothesized that DTT breaks disulfide bonds in assembly-incompetent tau dimers linked by a disulfide bond, thereby restoring the filament elongation that had been inhibited by these dimers [32]. According to this hypothesis, oligomers formed through intermolecular disulfide bonds between the three cysteine residues in MAP4 may inhibit the elongation of amyloid fibrils. These speculations suggests that *in vivo*, aggregates may be formed by the reduction of disulfide bonds in proteins due to abnormal physiological conditions such as cardiomyopathy or myocardial infarction, and similar considerations suggest that changes in the redox state of cells and tissues caused by reactive oxygen species that occur during aging or stress may result in abnormal disulfide bond formation in proteins [33].

Heparin has been used for many years as an inducer of tau protein aggregation, including in our recent work [16]. However, a recent study has shown that heparin-induced tau filaments are structurally distinct from those found in AD brains [34]. Although the structure of MAP4 aggregates *in vivo* is still unknown, the similarity of the amino acid sequences of these MAPs implies that the structures of heparin-induced aggregates and native aggregates *in vivo* are also different, as in the case of tau. This will be an important consideration for future research.

### 3.3 Comparison of MAP4 and tau aggregate process

**Fig. 3:**
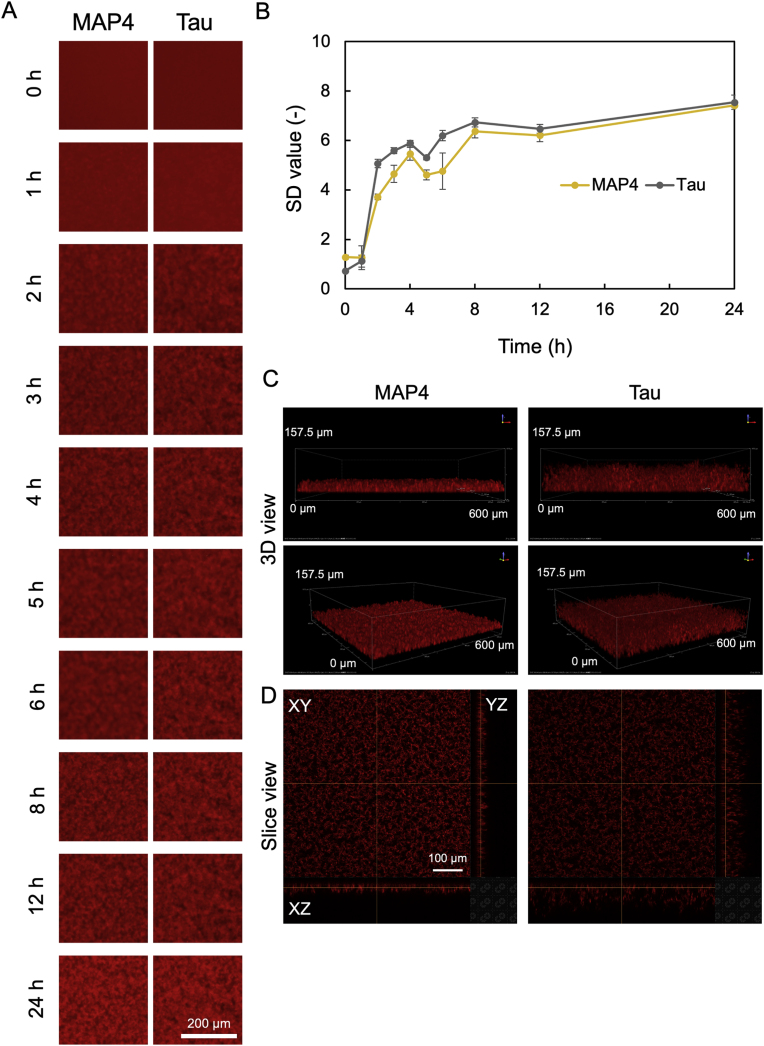
Comparison of MAP4 and tau aggregation rate. (A) Sequential images of the process of 10 μM MAP4 and tau aggregation. Images were captured at the indicated time point using conventional fluorescence microscopy for 24 h. (B) SD value at each time point was quantified using Image J software. Data represent mean values, and error bars indicate SDs derived from three separate experiments. (C) 3D reconstruction images of MAP4 and tau aggregates after 24 h incubation. (D) Slice images of aggregates of each sample in panel (C).

**Fig. 4:**
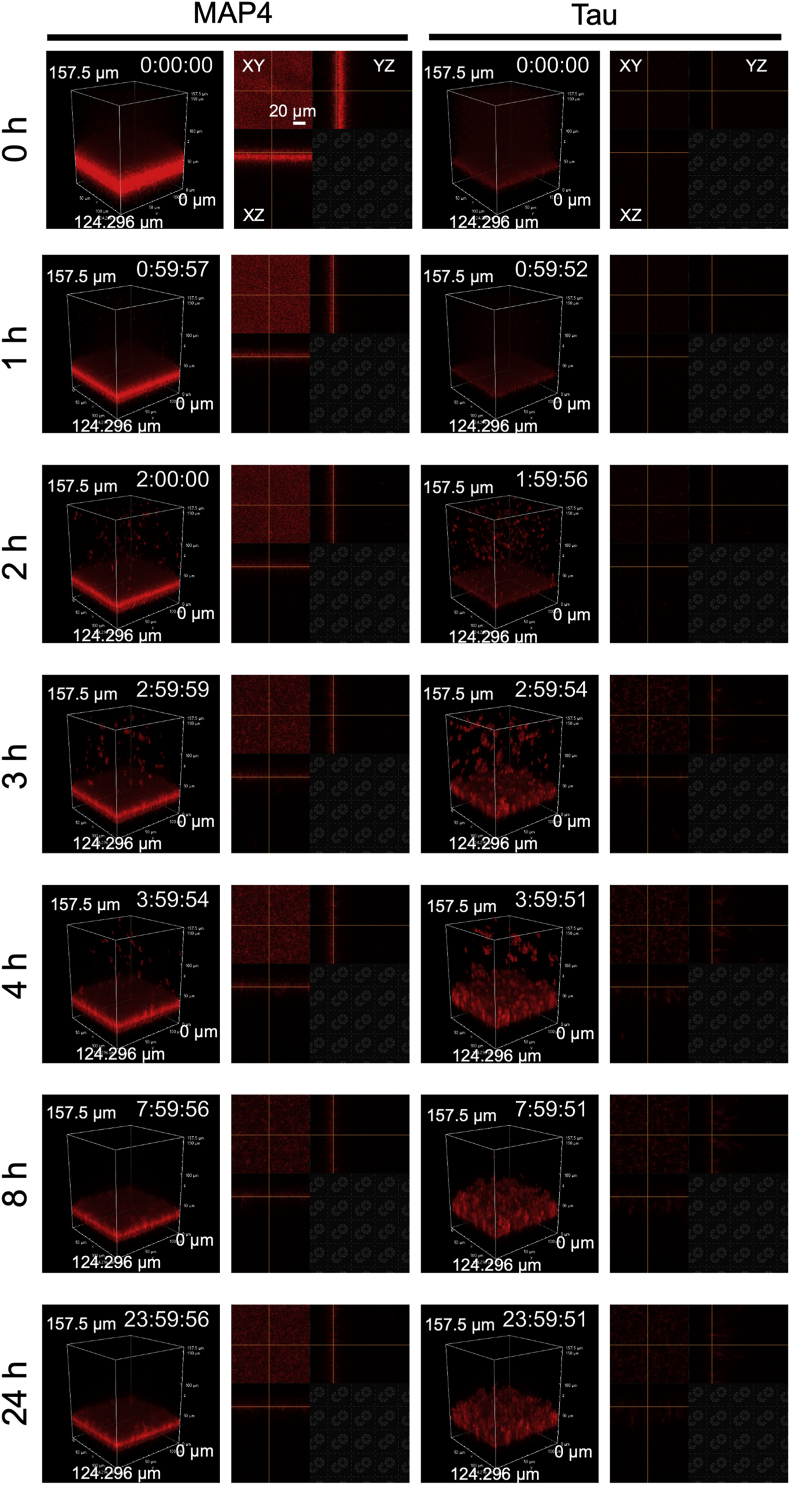
Comparison of MAP4 and tau aggregates. 3D reconstruction and slice images of MAP4 and tau aggregates at the indicated time points.

It was reported that MAP4 and tau exhibited similar primary structures of repeat regions [12]. To examine whether there are differences in the manner of aggregation between the two proteins, we observed the aggregation process in real-time using conventional microscopy and confocal laser microscopy. When 10 μM of MAP4 and tau were incubated at 37 °C in the presence of heparin and DTT, inverted fluorescence microscopy revealed that aggregates began to form after 1 h of incubation with both MAP4 and tau (Fig. 3A). Aggregate formation proceeded until 8 h of incubation in both cases, after which no changes in aggregates or SD values were observed (Fig. 3A and B). These results indicate that there is no difference in the rate of aggregate formation between MAP4 and tau. The 3D nature of aggregates at 24 h was observed by confocal laser microscopy (Fig. 3C and D). Interestingly, the aggregate thickness of MAP4 was smaller than that of tau, approximately 40 μm and 90 μm for 10 μM MAP4 and 10 μM tau, respectively. This was attributed to the fact that when MAP4 and tau formed aggregates in solutions and were observed to settle at the bottom of wells (Fig. 4 and Supplementary Movie S1 and Movie S2), MAP4 aggregate grains were smaller than those for tau. Therefore, the size of aggregate grains may have resulted in the smaller aggregate thickness of MAP4.

### 3.4 Detailed analysis of aggregate structures

**Fig. 5:**
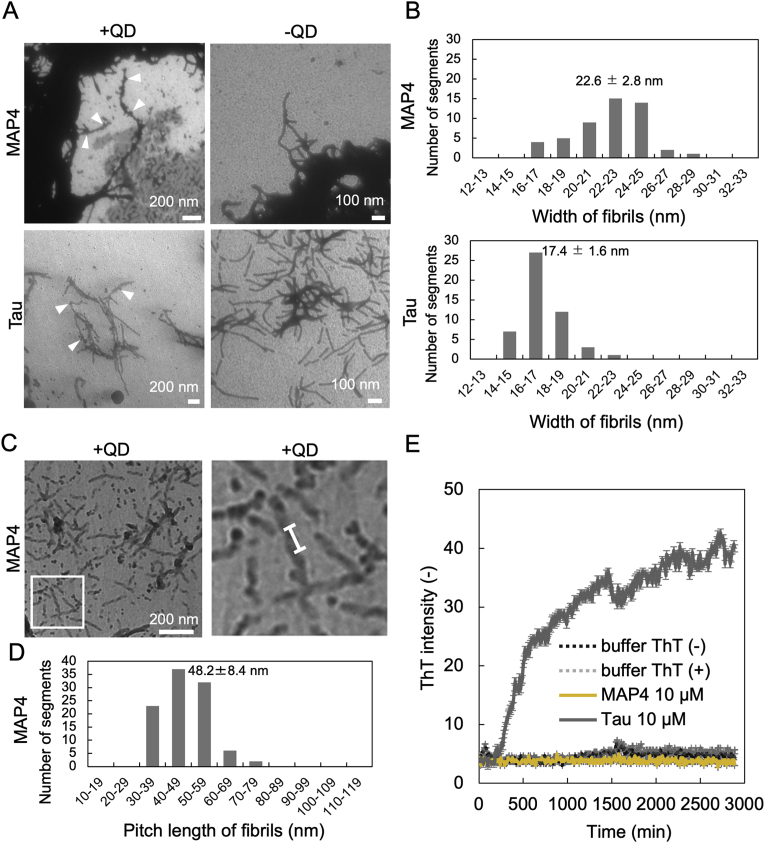
TEM observation and ThT assay for MAP4 aggregates. (A) TEM images of MAP4 and tau aggregates. The white arrow indicates the typical QDs. (B) Histograms of the width of each of the MAP4 and Tau fibrils based on images taken by TEM (n = 50 segments). (C) Figure on the left is high-resolution TEM images of MAP4 aggregates. Figure on the right is Enlarged high-resolution TEM images of aggregates of sample in panel C lower left white square. The white line indicates the length of the helical pitch. (D) Histograms of the helical pitch length of the MAP4 fibrils based on images taken by TEM (n = 100 pitches). (E) Monitoring ThT fluorescence intensity of MAP4 and tau aggregation. ThT intensity was measured every 10 min for 48 h. Data represent mean values, and error bars indicate SDs derived from three separate experiments.

As shown in Fig. 1, Fig. 2, Fig. 3, Fig. 4, we succeeded in observing MAP4 aggregation *in vitro*. Finally, to confirm whether these aggregates consist of amyloid fibrils, like tau aggregates, we observed them by TEM and assessed them with the ThT assay, in which 10 μM of MAP4 and tau was incubated at 37 °C in the presence of heparin and DTT, with or without QDs. The TEM observations revealed that MAP4 formed fibril structures with or without QDs, and QDs were bound to the formed fibrils (Fig. 5A). Similar to our previous reports, tau also formed fibril structures [16,35], and an analysis of the width of MAP4 and tau fibrils revealed that their widths were 22.6 ± 2.8 nm and 17.4 ± 1.6 nm, respectively (Fig. 5B). Tau was previously reported to form a Paired helical filament (PHF) with a width of 14.8 ± 0.6 nm [36], which is similar to the results of this experimental analysis. Further high-resolution observation of MAP4 fibrils revealed the fibrils have a helical structure and a helical pitch length of 48.2 ± 8.4 nm (Fig. 5C and D). These results suggested that MAP4 forms helical filaments similar to tau, although the helical morphology differs somewhat.

To confirm whether MAP4 aggregates included ThT-reactive β-sheet structures like tau, we performed the ThT assay using a microplate reader (Fig. 5E). The fluorescent dye ThT has become among the most widely used for selectively staining and identifying β-sheet rich amyloid fibrils both *in vivo* and *in vitro* [37]. The large enhancement of its fluorescence emission upon binding to amyloid fibrils makes ThT a particularly powerful and convenient tool [37]. On the other hand, studies with apolipoprotein E (ApoE) have reported that some α-helix rich fibrils show enhanced ThT fluorescence, so the results of the ThT assay should be interpreted with caution [38]. Conditions for the experiment were 10 μM MAP4 or tau, with or without 20 μM ThT in PBS containing 10 μM heparin and 10 mM DTT. The results showed that tau increased the fluorescence intensity of ThT, confirming the formation of amyloid fibrils [[39], [40], [41]]. However, MAP4 surprisingly did not increase ThT intensity. The ThT experiment is the gold standard for detecting amyloid fibrils, but is not absolute, because some amyloid fibrils do not show thioflavin fluorescence [42]. Several β-sheet-rich proteins did not show characteristic ThT fluorescence: transthyretin (TTR), chymotrypsin, bovine IgG, and concanavalin A [42]. Our results suggested that MAP4 formed helical filaments without ThT fluorescence. Other studies have shown that characteristic ThT fluorescence is induced in cavities long enough to accommodate the full length of ThT ions with diameters of about 8–9 Å in amyloid fibril structures, and that cavities small enough to bind a single ThT ion cannot provide an adequate binding environment to induce characteristic fluorescence [42]. The same study reported that a cavity small enough to bind a single ThT ion does not provide an adequate binding environment to induce characteristic fluorescence [42]. In this experiment, tau aggregates increased ThT fluorescence intensity, suggesting that MAP4 and tau belong to the same MAP family but have different aggregate structures. The results of the comparison of aggregates based on QDs suggest that the aggregate grains of MAP4 are smaller than those of tau (Fig. 3). Consequently, aggregate thickness is also smaller. The helical pitch length of MAP4 is 48.2 ± 8.4 nm from Fig. 5D, and the pitch length of Tau is reported to be 74 ± 8.5 nm in other papers [36], suggesting that MAP4 aggregates are still more densely structured than Tau. Taken together, these results suggest that MAP4 has smaller cavities in its amyloid fibril structure than tau, which is consistent with the fact that TTR and β-Cyclodextrin (β-CD) both have small cavities and cannot induce the characteristic fluorescence of ThT [42], further suggesting that the formation of dense aggregates may inhibit the induction of ThT fluorescence.

**Fig. 6:**
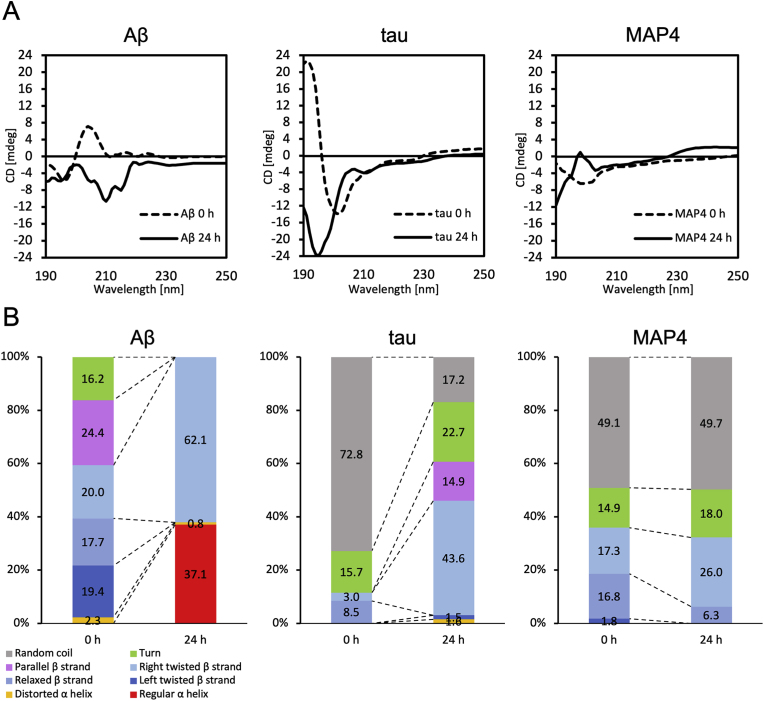
Assessment of secondary structure changes in of Aβ_42_, tau, and MAP4 during aggregation using CD spectroscopy. (A) CD spectra of each protein before (dashed lines) and after 24 h of aggregation (solid lines). (B) Proportions of secondary structure elements calculated using the BeStSel web server.

To investigate the secondary structure of MAP4 aggregates in more detail, we performed CD spectroscopy (Fig. 6). For comparison, we also performed measurements on Aβ_42_ and tau. Tau shows a characteristic CD spectrum with a prominent minimum at 198 nm and no significant signal at 220 nm [43]. In this study, tau showed a similar peak at 0 h, as did MAP4 (Fig. 6A). Quantification of secondary structure at 0 h and 24 h showed that the proportion of β-sheet structure increased at 24 h for Aβ and tau but decreased for MAP4. However, the proportion of right-twisted β-sheet structure characteristic of amyloid fibrils [44,45] increased in all proteins (Fig. 6B). Although further details are required, this result suggests that MAP4 exhibits a different β-sheet structure from tau, which may be reflected in the difference in the ThT assay (Fig. 5E).

In neurons of the brains of AD patients, hyperphosphorylated tau forms aggregates consisting of amyloid fibrils called paired helical filaments [46]. Abnormal "puncta" composed of phosphorylated MAP4 were also observed in post-ischemic mouse cardiomyocytes [8]. Although the relationship between phosphorylation and aggregation in these MAPs remains unclear, this study demonstrated that at least non-phosphorylated MAP4 forms aggregates consisting of helical filaments at a rate similar to that of tau. Future studies of the effects of phosphorylation and its impact on cellular homeostasis of their aggregates will shed light on how MAP4 and tau aggregates affect the pathogenesis of AD and heart failure.

## 4 Conclusion

In conclusion, we have shown that MBD fragments of MAP4 form aggregates composed of helical filaments on a time scale similar to that of tau. On the other hand, the size of aggregate grains, the thickness of the deposition, helical structure of filaments, and the sensitivity to ThT were different between MAP4 and tau. Similar to tau, MAP4 aggregates may disrupt cellular homeostasis, and elucidating this mechanism may be a promising therapeutic target for improving cardiac healing function in cardiac disease.

## CRediT authorship contribution statement

**Shuto Miura:** Writing – original draft, Formal analysis, Data curation. **Eisuke Ishibashi:** Formal analysis, Data curation. **Takuto Nakamichi:** Data curation. **Koji Uwai:** Formal analysis, Data curation. **Masahiro Kuragano:** Writing – review & editing, Supervision, Formal analysis. **Kiyotaka Tokuraku:** Writing – review & editing, Supervision, Project administration, Funding acquisition, Conceptualization.

## Declaration of competing interest

The authors declare that they have no known competing financial interests or personal relationships that could have appeared to influence the work reported in this paper.

## Data Availability

Data will be made available on request.
