# Supplementary material for: Microtubule-associated protein 4 forms aggregates consisting of helical filaments
Source: Biochem Biophys Rep. 2025 Jun 27;43:102118. doi: 10.1016/j.bbrep.2025.102118 (PMC12269874; doi:10.1016/j.bbrep.2025.102118)
Supplement: Multimedia component 1 [file mmc1.docx]

**Supplementary Materials**

***Biochemistry and Biophysics Reports***

Miura *et al.*

**Supplementary figures**


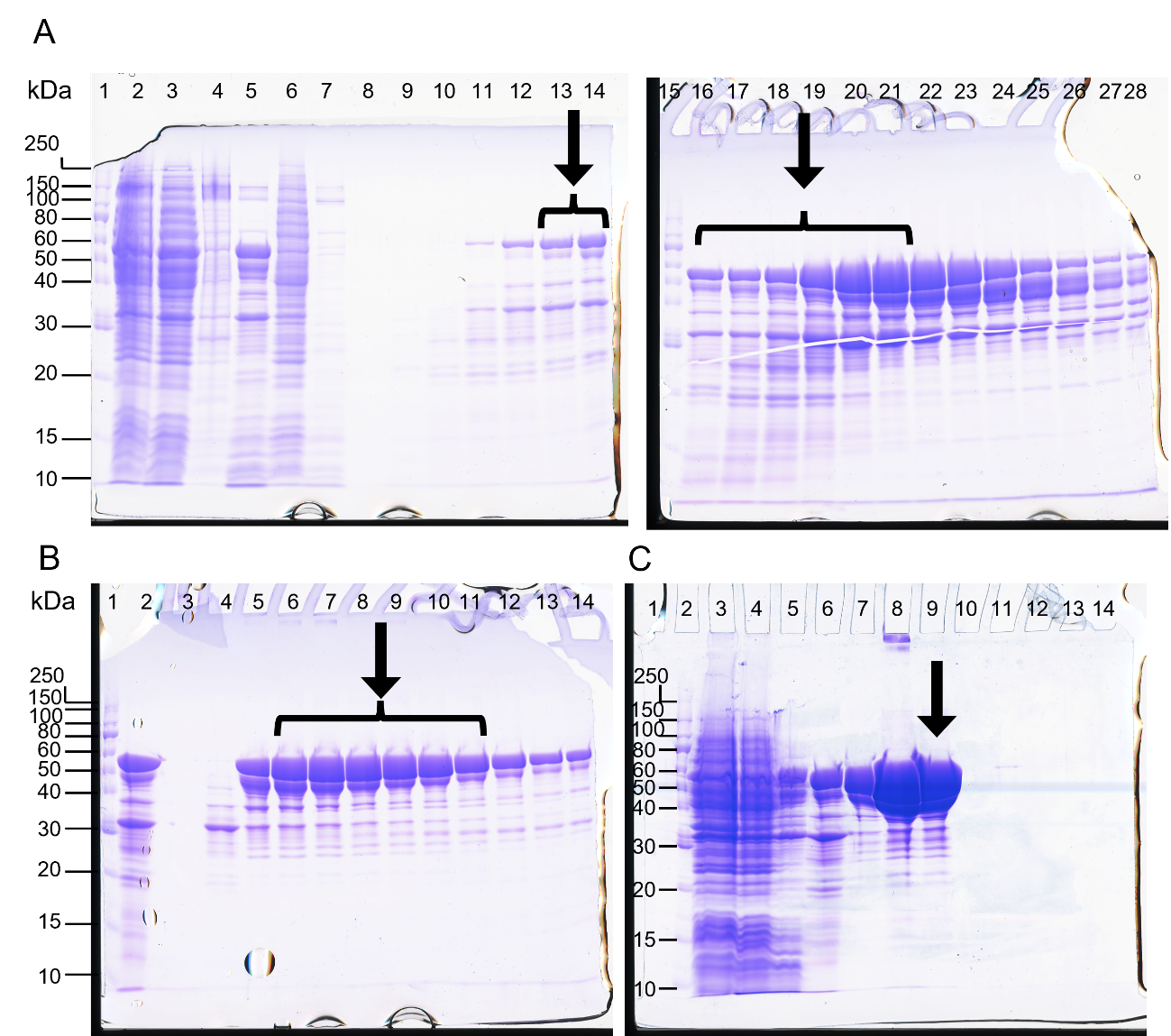


**Supplementary Fig. S1.** Electrophoretic patterns of purified human MAP4 MBD fragments. (A) Electrophoretic patterns of a phosphocellulose UNOsphere™ S column (Bio-Rad Laboratories Inc.). Lane 1 is the molecular weight marker (CLEARLY Protein Ladder, 3453A, Takara Bio Inc.), lane 2 is after sonication, lane 3 is supernatant after sonication, lane 4 is precipitate after sonication, lane 5 is supernatant after heat treatment, lane 6 is precipitate after heat treatment, lane 7 is UNO bare, lane 8 is UNO WASH, lanes 9-14 are fraction numbers 24-29, lane 15 is the molecular weight marker, and lanes 16-28 are fraction numbers 30-42. Black arrows indicate fractions used in the experiment. (B) Electrophoretic patterns of a TOYOPEARL® Butyl-650 column (Tosoh Co., Ltd.). Lane 1 is the molecular weight marker, lane 2 is UNO fraction collection, lane 3 is TOYO bare, lane 4 is TOYO WASH, and lanes 5-14 are fraction numbers 24-33. Black arrows indicate fractions used in the experiment. (C) Electrophoretic patterns of purified MBD fragments of MAP4. Lane 1 is empty, lane 2 is the molecular weight marker (CLEARLY Protein Ladder, 3453A), lane 3 is after sonication, lane 4 is supernatant after sonication, lane 5 is supernatant after heat treatment, lane 6 is UNO fraction collection, lane 7 is TOYO fraction collection, lane 8 is solution after centrifugal concentration, lane 9 is purified MAP4, lane 10 is empty, lane 11 is concentrated external solution, lane 12 is dialysis solution 1, lane 13 is dialysis solution 2, and lane 14 is dialysis solution 14. Black arrows indicate MAP4 used in the experiment.


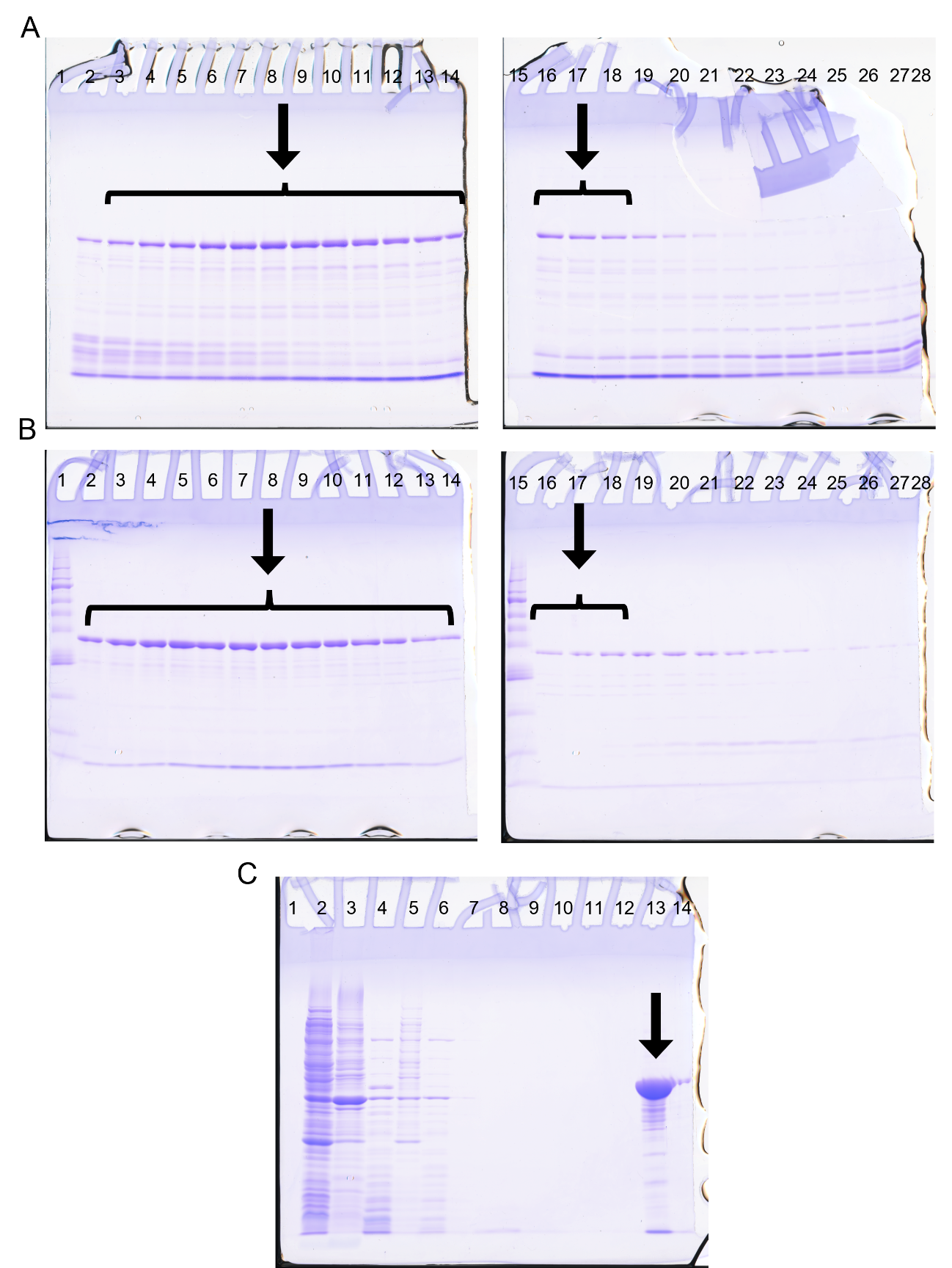


**Supplementary Fig. S2.** Electrophoretic patterns of purified human tau MBD fragments. (A) Electrophoretic patterns of a phosphocellulose UNOsphere™ S column (Bio-Rad Laboratories Inc.). Lane 1 is empty, lanes 2-14 are fraction numbers 33-45, lane 15 is empty, and lanes 16-28 are fraction numbers 46-58. Black arrows indicate the fraction numbers used in the experiment. (B) Electrophoretic patterns of a TOYOPEARL® Butyl-650 column (Tosoh Co., Ltd.). Lane 1 is the molecular weight marker, lanes 2-14 are fraction numbers 47-59, lane 15 is the molecular weight marker, and lanes 16-28 are fraction numbers 60-72. Black arrows indicate the fraction numbers used in the experiment. (C) Electrophoretic patterns of purified MBD fragments of tau. Lane 1 is empty, lane 2 is supernatant after sonication, lane 3 is precipitate after sonication, lane 4 is supernatant after heat treatment, lane 5 is precipitate after heat treatment, lane 6 is UNO bare, lane 7 is UNO WASH, lane 8 is TOYO bare, lane 9 is TOYO WASH, lane 10 is dialysis solution 1, lane 11 is dialysis solution 2, lane 12 is dialysis solution 3, lane 13 is purified Tau, and lane 14 is concentrated external solution. Black arrows indicate Tau used in the experiment.


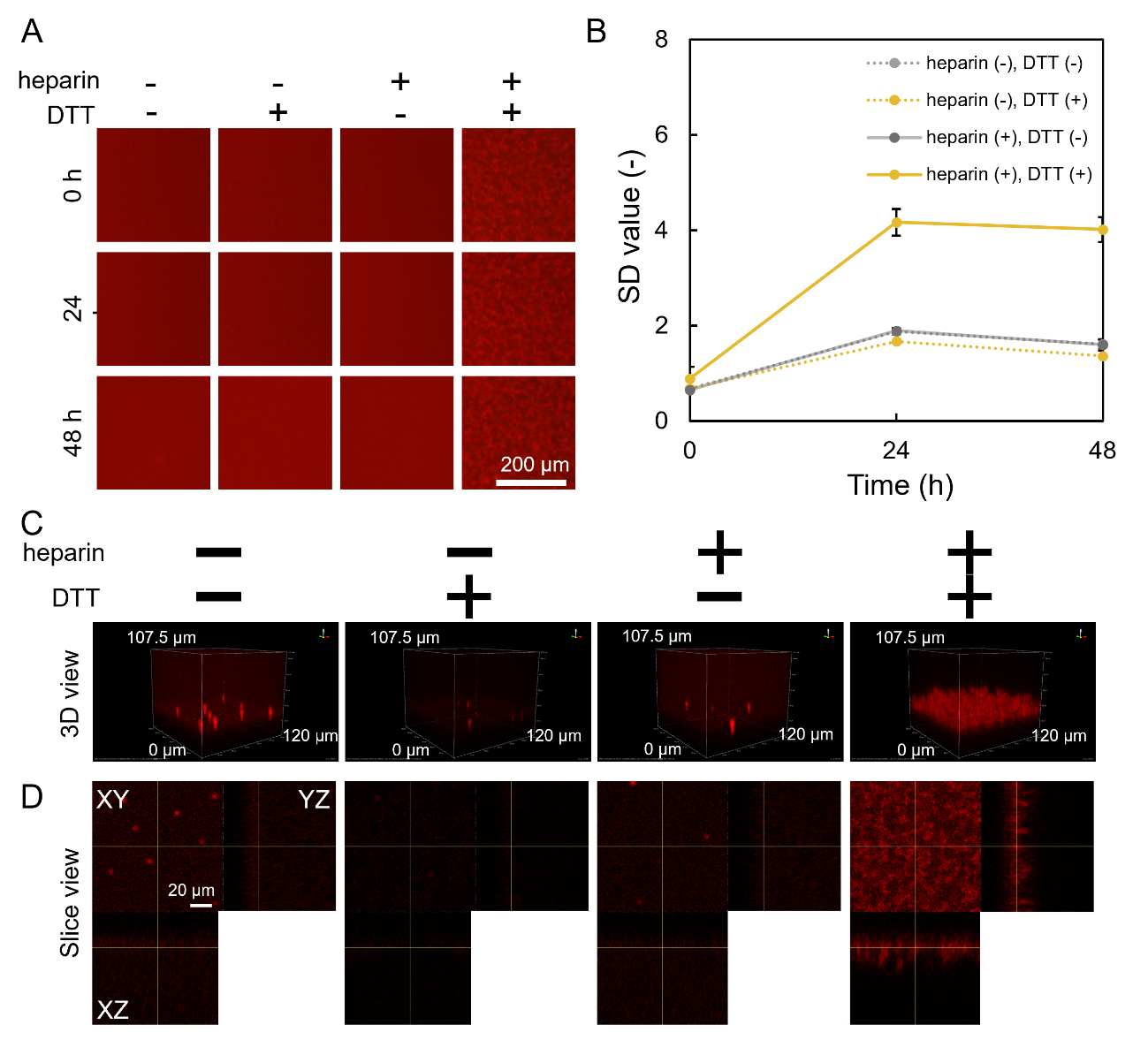


**Supplementary Fig. S3.** Assessing of the appropriate buffer condition for MAP4 aggregation. (A) Sequential images of the process of MAP4 aggregation on the indicated buffer condition. Images were captured every 24 h using conventional fluorescence microscopy for 48 h. (B) SD value at each time point was quantified using Image J software. Data represent mean values, and error bars indicate SD derived from three separate experiments. (C) 3D reconstruction images of MAP4 aggregates after 24 h of incubation. (D) Slice images of aggregates of each sample in panel (C).

**Supplementary movies**

Suppl. Movie S1. 3D reconstruction of the process of MAP4 aggregation. Images were captured using confocal microscopy. 10 µM MAP4, 50 nM QD, 10 µM heparin, 10 mM DTT, and 1× Phosphate-buffered saline (PBS) were coincubated at 37 ℃ for 24 h.

Suppl. Movie S2. 3D reconstruction of the process of Tau aggregation. Images were captured using confocal microscopy. 10 µM Tau, 50 nM QD, 10 µM heparin, 10 mM DTT, and 1× Phosphate-buffered saline (PBS) were coincubated at 37 ℃ for 24 h.
